# Ionic liquids as stabilisers of therapeutic protein formulations: a review of insulin and monoclonal antibodies

**DOI:** 10.1007/s12551-024-01261-y

**Published:** 2024-12-26

**Authors:** Samuel Tien, Veysel Kayser

**Affiliations:** Sydney School of Pharmacy, Faculty of Medicine and Health, The University of Sydney, Sydney, NSW 2006 Australia

**Keywords:** Ionic liquid, Thermal stability, Conformational stability, Insulin, Monoclonal antibody, Formulation of biologics

## Abstract

Therapeutic proteins such as insulin and monoclonal antibodies (mAbs) have become an essential part of the modern healthcare system and play a crucial role in the treatment of various diseases including cancer and autoimmune disorders. However, their long-term stability is a significant concern, affecting efficacy, shelf-life, and safety. Ionic liquids (ILs) have emerged as promising additives to enhance protein stability and address the aforementioned issues. Indeed, recent studies indicate that biocompatible ILs, particularly choline-based ILs, have significant potential to improve stability while preserving proteins’ functionality. For instance, choline valinate has been shown to increase the melting temperature of insulin by almost 13 °C (Judy and Kishore Biochimie 207:20-32, 2023), while choline dihydrogen phosphate has increased the melting temperature of trastuzumab by over 21 °C (Reslan et al. Chem Commun 54:10622-10625, 2018). However, it is worth noting that the use of some ILs introduces a complex trade-off: while they can increase thermal stability, they may also promote protein unfolding, thereby reducing conformational stability. Moreover, selecting the most suitable IL and its optimal concentration is challenging, as different protein formulations may exhibit varying effects. This review provides a comprehensive overview of the existing literature on ILs as stabilisers for insulin and mAbs, documenting specific IL-protein combinations and conditions to identify potential future stabilising agents for biologics in general.

## Introduction

In the last three decades, protein-based therapies, especially monoclonal antibodies (mAbs), have sparked a revolution in medicine, changing the way diseases are treated and managed. They have revolutionised treatment options across various diseases, including cancer, autoimmune disorders and metabolic disorders where they have become first-line therapies (Bajracharya et al. 2019). In 2023, eight out of the top ten best-selling drugs were protein-based medicines, highlighting their significant impact on the healthcare industry (Verdin 2024). The most common therapeutic proteins include mAbs, hormones, enzymes, coagulation factors and cytokines (Ebrahimi and Samanta 2023). These proteins are predominantly available in injection forms. This is primarily due to their specific characteristics, such as large molecular sizes and susceptibility to degradation in the gastrointestinal tract (Angsantikul et al. 2020; Curreri et al. 2023). A commonly observed issue with protein-based drugs is that they inherently tend to denature and aggregate. Aggregation primarily occurs due to self-association of unfolded or partially unfolded proteins. Additionally, proteins can undergo other degradation pathways, such as chemical degradation through oxidation and deamidation, or cross-linking, including the formation of disulfide bonds (Wang and Roberts 2018; Wang et al. 2010). Many environmental conditions promote self-association, including the presence of hydrophobic surfaces, agitation, low or high pH and elevated temperatures as well as high protein concentrations (Huus et al. 2005; Sluzky et al. 1991; Berenson et al. 2012, Kayser et al. 2011). Controlling protein aggregation is crucial, as it significantly disrupts protein structure and function, resulting in diminished therapeutic activity and potential immunogenicity (Kayser et al. 2011; Pham and Meng 2020). Therefore, maintaining protein conformation and structure during production, transport and long-term storage is essential for maintaining their biological activity.

We identified that the stability of insulin and mAbs has been extensively studied with ionic liquids (ILs):

### Insulin

**Fig. 1:**
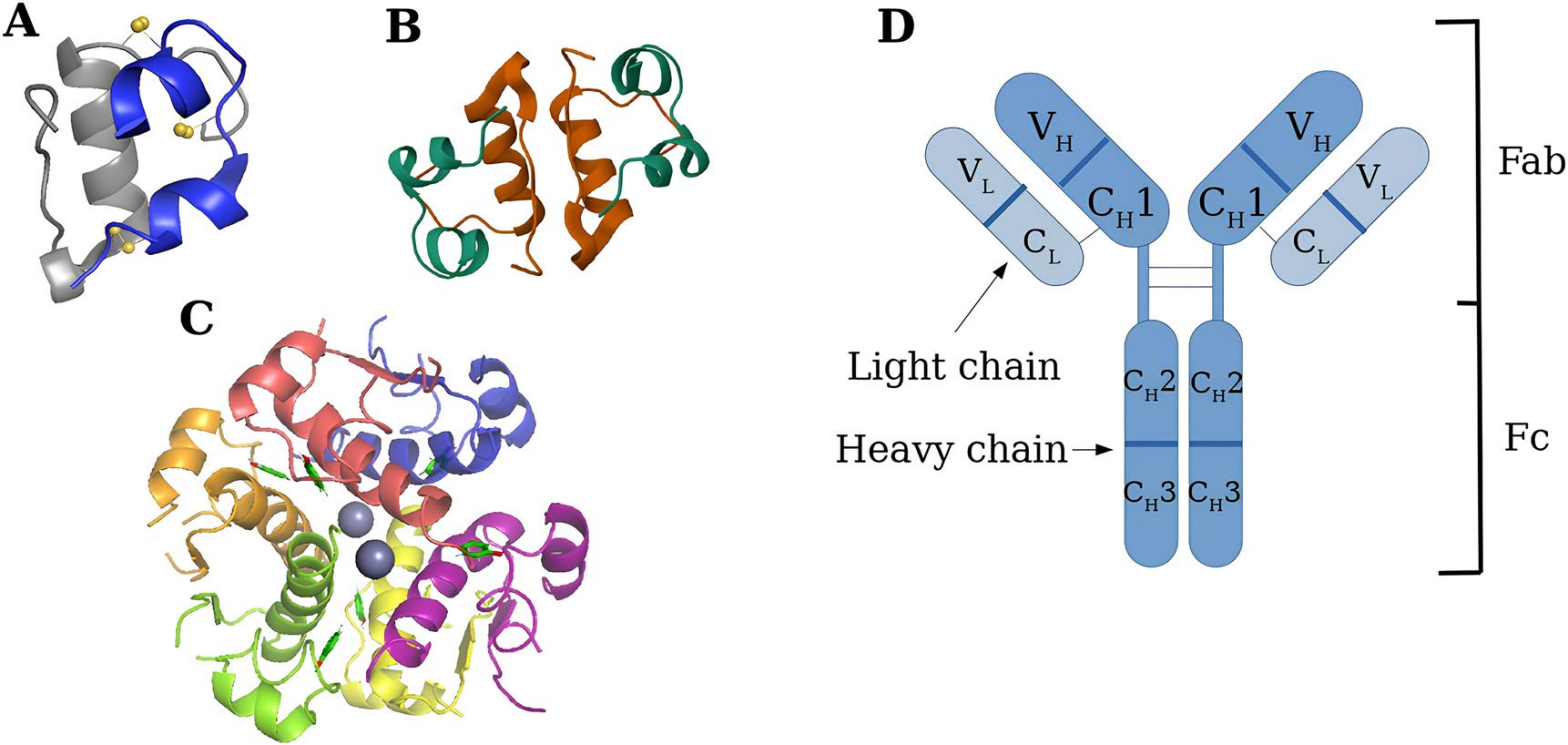
Structures of insulin and mAbs. **A** Human insulin monomer (RCSB: 5BTS) (Hjorth et al. 2016), showing disulfide bonds between A6–A11, A7–B7, and A20–B19. **B** Human insulin dimer (RCSB: 5BTS) (Hjorth et al. 2016). Contacts are between the B chains. **C** Human insulin hexamer in its most stable R6 conformation, formed around two zinc ions and stabilised by phenol molecules (RCSB: 1AI0) (Chang et al. 1997). **D** Structure of mAbs, with chains labelled

Insulin is a polypeptide hormone secreted by the beta cells of the pancreas that plays a crucial role in the homeostatic regulation of glucose metabolism. Ever since its discovery in 1916, insulin has remained an important therapeutic option for managing type I and type II diabetes mellitus (Gualandi-Signorini and Giorgi 2001). Initially, bovine and porcine insulins were used with varying degrees of success, as the structures were slightly deviated from human insulin. The advent of DNA recombinant technology in 1982 led to the production of the first commercially available human insulin formulation. Current insulin formulations are administered via subcutaneous injections using analogues that have distinct pharmacokinetic profiles. Rapid-acting insulins, which are aspart, lispro and glulisine, involve single amino acid substitutions, which enable them to mimic the rapid action of intravenous injections. Long-acting insulins, like glargine, detemir and degludec, incorporate fatty acid chains that bind to albumin, allowing for a slow, sustained release of the active monomer over time (Donnor and Sarkar 2023). Insulin is a relatively small protein with a molecular weight of 5808 Da. It is composed of an A chain (21 amino acid residues) and a B chain (30 amino acid residues), with one intrachain disulfide bond in chain A and two interchain disulfide bonds between chain A and chain B (Huus et al. 2005). At low concentrations, the protein is monomeric, which is the biologically active form (Fig. 1A). At the pH range of 2–8, low concentrations of phenol, or in millimolar concentrations, insulin associates into dimers connected by four hydrogen bonds (Fig. 1B) (Blundell et al. 1972; Maltesen et al. 2011). In the presence of bivalent ions like Zn^2+^ at neutral pH, insulin is assembled into hexamers (Fig. 1C). It is the hexamer structure that is the most stable; however, commercial insulin contains a mixture of all three forms in varying proportions (Mann et al. 2020).

### Therapeutic mABs

mAbs currently dominate the landscape of therapeutic protein drugs. As of November 2023, 130 different antibodies were in late-stage development, and 16 new therapies have been approved by regulatory agencies since the start of the year (Crescioli et al. 2024). In total, there are 5 classes of human antibodies: IgM, IgD, IgG, IgE and IgA. Currently, IgG is the only class utilised for therapeutic mAbs, which is further subdivided into IgG1-4. Among these, only IgG1, IgG2 and IgG4 have been employed in commercial mAbs. IgG3 is not used due to its extensive polymorphism, which could lead to inconsistent drug efficacy across different individuals, as well as its long hinge region, which is associated with reduced stability and aggregation propensity (Yu et al. 2020). IgGs are large ~ 150 kDa Y-shaped glycoproteins consisting of two heavy and two light polypeptides (Fig. 1D). In terms of activity, they are split into two distinct regions. The Fab region is responsible for binding to antigens. They have a constant and a variable domain on the light chains (CL and VL) and on the heavy chains (VH and CH1). Antibodies bind strongly to specific antigens, which is why they are used in a wide variety of applications where target specificity is preferred, such as oncology and immunology. The Fc region, containing two constant domains, CH2 and CH3, allows the molecule to interact with the immune system (Janeway et al. 2001). Variations in the Fc region allow for tuning of the serum half-life as well as the propensity for it to aggregate (Buss et al. 2012). Due to its large size, they are currently only available in the form of injections. While early mAb formulations were designed to be used intravenously, current formulations have shifted towards the subcutaneous route, mainly using prefilled syringes. This introduces new challenges, as these formulations typically require a small injection volume, necessitating very high protein concentrations and yet, minimal viscosity (Elgundi et al. 2017). Thus, special considerations must be taken in the choice and concentrations of the excipients. The following excipients are typically found in mAb formulations: a buffer (e.g. acetate, histidine) to maintain solution pH, salts (e.g. NaCl) to enhance solubility, a surfactant (e.g. polysorbate 20) to prevent aggregation, a stabiliser (e.g. sucrose) or a polyol (e.g. glycerol or sorbitol) to prevent aggregation and stabilise against stress, tonicity agents (e.g. mannitol) to maintain the osmotic balance, and enzymes (e.g. recombinant human hyaluronidase) to improve the permeation, absorption and dispersion of the injection (Mieczkowski 2023; Sifniotis et al. 2019).

### ILs

**Fig. 2:**
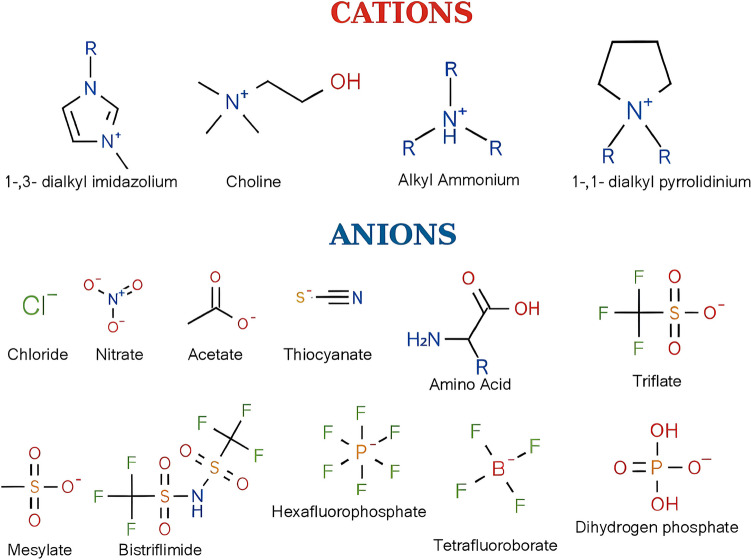
Examples of cations and anions in ionic liquids commonly used for pharmaceutical applications. Adapted from (Guncheva 2022)

ILs are molten salts composed of a cation and an anion with a melting point below 100 °C. They are characterised by their high ionic conductivity, low vapour pressure and low flammability (Schröder 2017). There are over 1 million combinations of cations and anions that can be tailored to fit into specific physicochemical properties, which has earned them the name “designer solvents” (Liu et al. 2010). The cations are typically organic and asymmetrical, while the anions are usually inorganic, with some exceptions including amino acids, mesylate, triflate and bistriflimide (Fig. 2) (Wilkes 2002; Guncheva 2022). They have been of interest recently due to their usage in catalysis, purification and electrochemistry (Liu et al. 2010; Pârvulescu and Hardacre 2007; Ventura et al. 2017), but most importantly due to their ability to stabilise proteins (Reslan and Kayser 2018; Fujita et al. 2006; Bharmoria and Kumar 2016).

The properties of ionic compounds and proteins in solution have been thoroughly investigated (Chen et al. 2007; Hribar et al. 2002; Galamba 2012) ever since their classification by Hofmeister (1888). The seminal paper ranked the ions by their ability to precipitate proteins. This effect is due to the ions’ ability to interact with the hydrogen-bonded network of water molecules surrounding the protein. The interaction is only limited to the first hydration shell (Reslan and Kayser 2018; Kang et al. 2020) but nevertheless can influence the protein conformation and protein–protein interactions. Ions that can cause a greater disruption to water are labelled as chaotropic, while those that bind strongly to water are kosmotropic (Kumar and Venkatesu 2014a). The effect of these ions on protein stability is mainly attributed to the anion, which tends to interact more strongly with the hydration shell (Reslan and Kayser 2018; Kang et al. 2020). Kosmotropic ions like dihydrogen phosphate, sulfate and hydroxide have larger charge densities. They reduce the protein’s interaction with water, which stabilises the conformation of the protein. Chaotropic ions like bromide, nitrate and thiocyanate disrupt the water-protein interface and protein–protein interactions. These ions enhance the solubility of the protein while also promoting its unfolding (Clark et al. 2020). The chloride anion is neither kosmotropic nor chaotropic. It is of note that protein interactions are complex, and the Hofmeister series has been found to not universally apply in all cases (Kumar and Venkatesu 2014a)**.**

Although some proteins can be fully dissolved in pure ILs, formulations typically include an aqueous solution such as water because of higher solubility, stability and reduced costs (Schröder 2017). Pure ILs also have high viscosity (Yu and Chen 2021) which is a problem when it comes to injections. Most classes of ILs are cytotoxic, which is predominantly influenced by the cation, though choice of anion can still have drastic effects (Tzani et al. 2022). The major cytotoxic cations include imidazolium, pyridinium and phosphonium. Toxicity is associated with increasing alkyl chains and increasing concentrations (McLaughlin et al. 2021; Rengstl et al. 2014). Recent efforts have focused on developing “green” biocompatible ILs, which are less harmful to the environment and human health while maintaining the same versatility (Santiago et al. 2022; Carreira et al. 2021). These ILs mainly consist of choline cations (Tzani et al. 2022, Miao et al. 2022), inorganic anions like chloride and nitrate, or derivatives from organic acids, natural sugars, amino acids, terpenes and purines (Carreira et al. 2021). The extent to which ILs stabilise proteins is highly dependent upon the type of formulations, with three main factors:

1. The combination of the IL and protein. For example, [BMIM][SCN] was found to increase aggregation of cytochrome c, myoglobin, lysozyme, ribonuclease A and β-lactoglobulin (Takekiyo et al. 2015, 2017), but suppressed amyloid formation in insulin (Takekiyo and Yoshimura 2018).
2. The concentration of the IL and protein. As an example, SOD1 showed increased thermal stability with low concentrations of [Chol][Cl]. However, when increasing the concentration of the IL, the thermal stability is reduced (Meena et al. 2022).
3. Inclusion of buffer, pH and other excipients. For instance, the aggregates formed by α-lactalbumin in [BMIM][CH_3_SO_4_] were demonstrated to be pH dependent in the presence of either glycine or Tris–HCl buffer (Bae et al. 2011).

Despite promising findings, the interactions between proteins and ILs are still not fully understood and require thorough, case-by-case analysis. In this review, we examine various studies on the impact of ILs on insulin and mAbs. Our analysis reveals that ILs affect both conformational and colloidal stability. Conformational stability refers to preserving the protein’s native shape, which is crucial for preserving its function. Colloidal stability pertains to the protein’s tendency to self-interact and aggregate, often evaluated through long-term storage studies, or in accelerated degradation experiments under conditions like elevated temperatures or in the presence of denaturants. It is of note that these two aspects are interconnected; for example, partial unfolding of proteins often precedes aggregation. Although counterintuitively, it is also possible to lower conformational stability while increasing colloidal stability, and vice versa, as seen below. These two key factors will be highlighted in a summary table.

## Stability of insulin with ionic liquids

### Choline-based ionic liquids

Choline cations are considered the main class of biocompatible ILs. Most of the studies investigating the stability of insulin formulations use amino acid anions. This is most likely because they are biocompatible and biodegradable, and have low toxicity (Le Donne and Bodo 2021; Hou et al. 2013; Foulet et al. 2016). Table 1 summarises the findings for the ILs discussed below.

**Table 1:** Summary of insulin stability in choline-based ILs

Ionic liquid	IL concentration	Colloidal stability	Conformational stability
5 μM human recombinant insulin in 20 mM phosphate buffer at 2 pH (Judy and Kishore 2023)			
[Chol][Gly]	2.5 mM	+	/
	10 mM	+	/
	50 mM	+	-
[Chol][Leu] [Chol][Val] [Chol][Pro]	2.5 mM	+	/
	10 mM	+	-
	50 mM	+	-
0.517 mM porcine insulin in 10 mM KCl/HCl buffer at 2 pH (Guncheva et al. 2019)			
[Chol]_2_[Asp]	0.766 mM	+	-
[Chol]_2_[Glu]	0.766 mM	-	-
[Chol][Arg]	0.766 mM	-	+
[Chol][Lys]	0.766 mM	-	+
[Chol][Asp]	0.766 mM	+	N/A
[Chol][Glu]	0.766 mM	+	-
0.5 mg/mL insulin aspart in 10 mM phosphate buffer at 7.4 pH (Sundaram et al. 2022)			
[Chol][Pro]	50–1000 mM	N/A	+
[Chol][Gly]	50–1000 mM	N/A	-
[Chol][Ala]	50–1000 mM	N/A	-
Insulin in pure choline geranate (CAGE) (Banerjee et al. 2018)			
[Chol][Geranate]	Pure	+	+

+ = increased stability, / = no change, - = decreased stability

Judy and Kishore (2023) studied the stability of [Chol][Gly], [Chol][Leu], [Chol][Pro] and [Chol][Val] on human recombinant insulin. Through accelerated studies at higher temperatures, all the ILs at concentrations of 2.5–50 mM enhanced the thermal stability of insulin compared to the non-IL sample. Notably, [Chol][Val] exhibited the highest increase in melting temperature to 71 °C, from a control of 58.4 °C. Increasing the concentrations of ILs led to a reduction in the formation of protein aggregates, with complete inhibition of amyloid and fibril formation observed at a concentration of 50 mM. The resistance to amyloid formation was as follows: [Chol][Gly] > [Chol][Pro] > [Chol][Leu] > [Chol][Val]. In terms of conformational stability, for [Chol][Gly], β-sheet formation was observed at 2.5 mM; however, the protein maintained high thermal stability at concentrations above 10 mM. In [Chol][Leu], [Chol][Pro] and [Chol][Val], the protein underwent a transition to random coil structures at 2.5 mM and 10 mM, but at a concentration of 50 mM, ILs completely suppressed these formations. Conformational stability decreased as the concentration of the ILs increased. [Chol][Leu], [Chol][Pro] and [Chol][Val] slightly altered the α-helix backbone of the insulin at 1 mM, while severely disrupting the backbone at concentrations of 10 mM and 50 mM. This disruption can be attributed to the direct interaction between the IL and the protein, as well as its ability to reduce hydration around the insulin, leading to increased misfolding. In the case of [Chol][Gly], the α-helix backbone was preserved at concentrations ranging from 1 to 10 mM and disrupted at 50 mM. This is hypothesised to be due to its lower hydration number compared to other amino acids tested, allowing insulin to maintain a higher degree of interface with water. Among the tested ILs, [Chol][Gly] at concentrations of 10 mM and below exhibited the highest potential by significantly enhancing colloidal stability while preserving conformational stability.

Guncheva et al. (2019) studied the stability and the toxicity of [Chol][Glu], [Chol][Asp], [Chol]_2_[Glu], [Chol]_2_[Asp], [Chol][Lys] and [Chol][Arg] on porcine insulin. The cytotoxicity of the ILs was evaluated at 0.05–5 mM with the 3T3 cell line. The order from least to most cytotoxic was found to be: [Chol][Asp] > [Chol]_2_[Asp] > [Chol][Lys] > [Chol]_2_[Glu] > [Chol][Glu] > [Chol][Arg]. Lower concentrations of all ILs at 0.05 mM and 0.5 mM had minimal effect on proliferation. However, at 5 mM, there was a significant reduction of cell growth. [Chol][Asp] was the least cytotoxic while [Chol][Arg] was the most, maintaining ~ 95% and ~ 55% cell viability respectively. The thermal stability of porcine insulin was assessed for ILs at a concentration of 0.776 mM. The order of highest to lowest thermal stability is as follows: [Chol][Glu] > [Chol]_2_[Asp] > [Chol][Asp] > IL-free > [Chol][Lys] > [Chol]_2_[Glu] > [Chol][Arg]. [Chol][Glu] displayed the highest stability, increasing the melting temperature from 75.4 to 85.1 °C. Interestingly, [Chol][Asp] displayed a more complex denaturing pathway, showing two different unfolding processes at 79.2 °C and 84.7 °C. Conformational stability was evaluated by analysing its secondary structures. The order of decreasing helical content is as follows: [Chol][Arg] > [Chol]_2_[Glu] > [Chol][Lys] > [Chol][Glu] > IL-free > [Chol]_2_[Asp]. [Chol][Glu] had higher helical content compared to the IL-free sample; however, it also displayed a greater degree of unfolded structures. [Chol][Arg], [Chol]_2_[Glu] and [Chol][Lys] demonstrate the highest conformational stability, with [Chol]_2_[Glu] adopting the most compact structure; however, its colloidal stability and toxicity profiles pose challenges for protein formulations.

Sundaram et al. (2022) studied the structure and interactions of insulin aspart with 50 mM, 500 mM and 1000 mM [Chol][Pro], [Chol][Gly] and [Chol][Ala] using both molecular dynamics simulations and physical experiments. Choline primarily interacted with the protein via Van der Waals forces and π-alkyl interactions with hydrophobic residues like phenylalanine. On the other hand, the amino acid anions interacted with the protein through hydrogen bonding, resulting in a more noticeable impact on the protein’s secondary structure. [Chol][Pro] demonstrated significant interactions with water, creating a hydrophobic environment surrounding the protein. Conversely, [Chol][Gly] and [Chol][Ala] exhibited the opposite effect, enhancing the hydrophilic nature. With increasing concentrations, [Chol][Pro] exhibited a higher amount of highly organised monomeric structures. Conversely, [Chol][Gly] and [Chol][Ala] tended to form aggregates at higher concentrations. These results suggest that the protein-water interactions contribute greatly to the denaturation of insulin aspart. Since prolinate is a kosmotropic ion, it is hypothesised that by reducing protein-water interactions as [Chol][Pro] does, the conformational stability is enhanced.

Banerjee et al. (2018) developed an oral insulin dosage form using choline geranate (CAGE), which demonstrated effective absorption through intestinal membranes. The increase in absorption is attributed to the ability of ILs to modulate intestinal mucus properties (Peng et al. 2021). Although the topic of IL’s effects on membrane interfaces is beyond the scope of this review, it is an interesting IL-protein application, which represents another promising pathway to explore. In this paper, they also assessed the long-term stability of insulin in pure CAGE solvent. Their findings indicated that insulin remained stable at room temperature (25 °C) for up to 2 months and under refrigeration (4 °C) for up to 4 months, with the α-helix structure of the protein well preserved in both conditions. The efficacy of insulin after storage in CAGE was further validated through animal studies, where there were minimal differences compared to the fresh sample. Additionally, a Caco-2 cell culture assay was employed to assess the cytotoxicity of CAGE at concentrations ranging from 10 to 50 mM, which showed a correlation between increasing CAGE concentrations and decreasing cell viability.

### Imidazolium-based ionic liquids

Imidazolium-based ILs represent one of the most extensively studied and well-established classes of ILs. Although imidazolium ILs are generally chaotropic and have been known to induce protein denaturation (Guncheva 2022; Hribar et al. 2002), understanding their effects on protein structure and stability is essential for advancing the knowledge of protein-IL interactions. The existing studies investigating insulin stability with imidazolium-based ILs have focused exclusively on the use of the 1-butyl-3-methylimidazolium (BMIM) cation. Table 2 summarises the findings for the ILs discussed below.

**Table 2:** Summary of insulin stability in imidazolium-based ILs

Ionic liquid	IL concentration	Colloidal stability	Conformational stability
0.43 M porcine insulin in 10 mM KCl/HCl buffer at 2 pH (Todinova et al. 2016)			
[BMIM][CH_3_COO]	300 mM	+	-
[BMIM][CF_3_COO]	300 mM	+	-
[BMIM][C(CN)_3_]	300 mM	-	-
[BMIM][N(CN)_2_]	300 mM	+	-
[BMIM][SCN]	300 mM	/	-
[BMIM][Cl]	300 mM	/	-
0.5 mg/mL zinc-free insulin in phosphate buffer at 7 pH (Kumar and Venkatesu 2014b)			
[BMIM][Cl]	10–40 mM	-	+
[BMIM][Br]	10–40 mM	-	+
[BMIM][SCN]	10–40 mM	-	-
[BMIM][HSO_4_]	10–40 mM	-	-
[BMIM][CH_3_COO]	10–40 mM	-	-
[BMIM][I]	10–40 mM	-	-

+ = increased stability, / = no change, - = decreased stability

One interesting property of ILs is that they encourage the formation of reversible aggregates when stressed, which can then be reformed back into their native structure. This was investigated by Todinova et al. (2016). [BMIM][CH_3_COO], [BMIM][Cl], [BMIM][CF_3_COO], [BMIM][SCN], [BMIM][N(CN)_2_] and [BMIM][C(CN)_3_] were tested for porcine insulin. Thermal stability in 300 mM of these ILs was assessed as follows: [BMIM][CH_3_COO] > [BMIM][CF_3_COO] > [BMIM][N(CN)_2_] > [BMIM][Cl] = IL-free > [BMIM][SCN] > [BMIM][C(CN)_3_]. The ILs [BMIM][CH_3_COO] and [BMIM][CF_3_COO] both increased thermal stabilities significantly, increasing the melting temperatures to 86.85 °C and 86.59 °C respectively, from the control of 75.43 °C. Interestingly, the cyano-containing anions deviates from the Hofmeister series. Typically, as the number of cyano-anions increases, the ion becomes more chaotropic, which should decrease thermal stability (Batista et al. 2015). However, [BMIM][N(CN)_2_] unexpectedly displays higher thermal stability than [BMIM][SCN], which indicates the involvement of more complex interactions. [BMIM][CH_3_COO] exhibited 90% reversibility of its aggregates. [BMIM][CF_3_COO] and [BMIM][SCN] displayed partial reversibility while [BMIM][N(CN)_2_], [BMIM][C(CN)_3_] and [BMIM][Cl] formed irreversible aggregates. In terms of conformational stability, both [BMIM][CH_3_COO] and [BMIM][CF_3_COO] showed excellent ability to preserve the secondary structure, while [BMIM][C(CN)_3_] had the most significant structural changes and promoted denaturation. Overall, colloidal stability is correlated with conformational stability for these imidazolium ILs.

Kumar and Venkatesu (2014b) investigated the effect of [BMIM][Br], [BMIM][Cl], [BMIM][HSO_4_], [BMIM][SCN], [BMIM][CH_3_COO] and [BMIM][I] in zinc-free insulin. Every single IL at a concentration of 10–40 mM reduced thermal stability in the following order: IL-free > [BMIM][Cl] > [BMIM][Br] > [BMIM][CH_3_COO] > [BMIM][SCN] > [BMIM][HSO_4_] > [BMIM][I]. As the concentration increases, a general trend of decreasing thermal stability can be observed. This effect is particularly pronounced with [BMIM][I], where at a concentration of 20 mM, the melting temperature decreased to 35.4 °C from the control of 84.2 °C. Even the relatively better performing ILs in low concentrations like 10 mM [BMIM][Cl] and [BMIM][Br] decreased the melting temperature significantly to 63.3 °C and 62.4 °C respectively. Conformational stability was assessed in 0.5–2.0 M of ILs. [BMIM][HSO_4_], [BMIM][SCN], [BMIM][CH_3_COO] and [BMIM][I] completely denatured the protein. Contrastingly, [BMIM][Br] and [BMIM][Cl] stabilised the structure. This observed effect of ILs on thermal stability is contrary to what is predicted by the Hofmeister series, as the sulfate anion was found to be more denaturing than chloride and bromide. Overall, [BMIM][Cl] and [BMIM][Br] were the best at stabilising the conformational structure of insulin; however, they also decreased the colloidal stability to a high degree.

### Alkylamine-based ionic liquids

Alkylamine-based ILs represent a large selection of cations. By varying the length of their alkyl chains, the physicochemical characteristics can be tailored to suit specific applications (Hoque et al. 2018). Table 3 summarises the findings for the ILs discussed below.

**Table 3:** Summary of insulin stability in alkylamine-based ILs

Ionic liquid	IL concentration	Colloidal stability	Conformational stability
1 mg/mL zinc-free insulin in 10 mM Tris buffer at pH 7 (Kumar and Venkatesu 2013)			
TMAS	0.5–2 M	/	+
TEAS	0.5–2 M	/	+
TMAP	0.5–2 M	+	+
TEAP	0.5–2 M	+	+
TMAA	0.5–2 M	+	+
1 mg/mL bovine insulin with 500 mM NaCl and 50 mM DCl at pD 1.6. (Takekiyo et al. 2016)			
[EAN][NO_3_]	10–30 mol%	+	+
[PAN][NO_3_]	10–30 mol%	+	+

+ = increased stability, / = no change, - = decreased stability

Kumar and Venkatesu (2013) investigated the effect of TMAS *([TMA][HSO*_*4*_*])*, TEAS *([TEA][HSO*_*4*_*])*, TMAP *([TMA][H*_*2*_*PO*_*4*_*])*, TEAP *([TEA][H*_*2*_*PO*_*4*_*])* and TMAA *([TMA][CH*_*3*_*COO])* on zinc-free insulin. Thermal stability was assessed using high IL concentrations of 0.5–2 M. The order of stability observed was [TEAP] > [TMAA] = [TMAP] > [TMAS] = [TEAS] = IL-free. 2 M TEAP displayed the greatest thermal stability, increasing the melting temperature to 79.3 °C from the control of 63.9 °C. As a general trend, higher concentration was correlated with higher thermal stability. In the presence of these ILs, the insulin adopts a more compact structure and is more ordered. From highest to lowest conformational stability is as follows: TEAP > TMAA = TMAP > TMAS > TEAS, which directly correlates to thermal stability. The hydrodynamic radius of the insulin is drastically reduced in all concentrations of ILs. These protic ILs were found to interact unfavourably with the surface of the protein and increase stability without altering any of the functional groups. All in all, TEAP, TMAA and TMAP are very promising ILs as they increase thermal stability while stabilising the secondary structure.

Takekiyo et al. (2016) compared the activity of 10–30 mol% alkylamine ILs [EAN][NO_3_] and [PAN][NO_3_] to the imidazolium IL [BMIM][SCN] on bovine insulin. All the ILs showed a significant suppression of amyloid formation above 10 mol% under high temperatures. In contrast to [EAN][NO_3_] and [PAN][NO_3_], which almost exactly matched monomeric insulin in these conditions, [BMIM][SCN] exhibited a higher proportion of unfolded structures and a reduced content of α-helical structures. Similarly, the conformational stability was greater for alkylamine ILs. [PAN][NO_3_] showed the least proportion of amyloid formation at 0.57%, followed by [EAN][NO_3_] at 0.67% and [BMIM][SCN] with 6.8%. Hence, the tested alkylammonium ILs exhibit greater overall stability compared to the imidazolium IL [BMIM][SCN].

## Stability of monoclonal antibodies with ionic liquids

In the existing literature, only choline-based ILs have been investigated in combination with mAbs. This can be attributed to their demonstrated efficacy in stabilising various smaller proteins and their non-toxic nature, as previously discussed. [Chol][Dhp] was the primary IL of interest in these studies, and surprisingly, there were no studies with amino acid anions. Most of these studies had a large focus on investigating properties with other excipients as well as long-term storage. Table 4 summarises the findings for ILs with mAbs.

**Table 4:** Summary of the stability of monoclonal antibodies with ILs

Ionic liquid	IL concentration	Colloidal stability	Conformational stability
Unbuffered 20 mg/mL trastuzumab (Reslan et al. 2018)			
[Chol][Dhp]	25% w/v	/	N/A
[Chol][Dhp]	40% w/v	+	N/A
[Chol][Dhp]	50% w/v	+	N/A
60 mg/mL trastuzumab in 5.5 mM l-histidine, 6.4 mM l-histidine HCl monohydrate, 144 mM trehalose dihydrate and 0.001% w/v polysorbate 20 (Reslan et al. 2018)			
[Chol][Dhp]	30% w/v	+	N/A
[Chol][Dhp]	53% w/v	+	N/A
EGFR mAb in PBS buffer with 20 μg/mL proteinase K at 37 °C (Mazid et al. 2015)			
[Chol][Dhp]	20% w/w	+	-
[Chol][Dhp]	50% w/w	+	-
1.5 μM IgG in 10 mM sodium phosphate buffer at pH 7 (Dhiman et al. 2022)			
[Chol][CH_3_COO]	0.5–2.5 mM	+	/
[Chol][Cl]	0.5–2.5 mM	+	/
[Chol][Dhc]	0.5–2.5 mM	+	/
[Chol][Dhp]	0.5–2.5 mM	+	/
Unbuffered 10 mg/mL IgG4 (Shmool et al. 2021)			
[Chol][Dhp]	10% w/w	-	-
100 mg/mL IgG4 in 34 mg/mL l-arginine HCl, 50 mg/mL trehalose dihydrate and 0.49 mg/mL polysorbate 20 (Shmool et al. 2021)			
[Chol][Dhp]	10% w/w	-	+
50 mg/mL IgG4 in 34 mg/mL l-arginine HCl, 50 mg/mL trehalose dihydrate and 0.49 mg/mL polysorbate 20 (Schmool et al. 2021)			
[Chol][Dhp]	10% w/w	-	-
20 mg/mL IgG4 in water. Results from fresh samples and samples stored at 4 °C for 365 days (Shmool et al. 2022)			
[Chol][Cl]	10–50% w/w	+	+

+ = increased stability, / = no change, - = decreased stability

Reslan et al. (2018) investigated the stability of trastuzumab in the presence of [Chol][Dhp]. The ILs were tested in an unbuffered solution with 20 mg/mL of trastuzumab and then at 60 mg/mL with the regular excipients in the Herceptin® formulation: 5.5 mM l-histidine, 6.4 mM l-histidine HCl monohydrate, 144 mM trehalose dihydrate and 0.001% w/v polysorbate 20. Thermal stability was assessed with 25%, 40%, and 53% w/v [Chol][Dhp]. In the unbuffered sample, at 25% w/v IL, [Chol][Dhp] was found to have a similar effect to 15 mM potassium phosphate buffer. In these samples, two distinct stages of denaturation occurred, corresponding with first; the unfolding of the CH2 domain, and second; the Fab and the CH3 domain unfolding simultaneously. Interestingly, at higher concentrations of [Chol][Dhp] at 40% and 53% w/v, a distinct three-stage unfolding process was observed, corresponding with the second stage of unfolding being split into two events. It appears that one domain is substantially more stabilised than the other, leading to the events taking place at different temperatures. In the 60 mg/mL sample at 30% w/v of IL, a similar unfolding behaviour to the control was observed, with two distinct stages of unfolding at melting temperatures Tm1 and Tm2. The IL increased Tm1 to 77.8 °C from 72.9 °C but had a minimal effect on Tm2, which increased slightly from 89.7 °C compared with the control of 88.2 °C. The 53% w/v IL concentration exhibited a single thermal unfolding event at 94 °C indicating a very high level of thermal stability. More critically, the aggregates formed in 53% w/v IL were found to be reversible, as indicated by the significant presence of monomers after the heating process. It was also seen that while the IL increased the temperature for the initial monomers to aggregate, once those temperatures were reached, the process of aggregation occurred at a faster rate. Overall, [Chol][Dhp] demonstrates a notable increase in thermal stability, and this effect is further amplified when combined with other excipients.

Mazid et al. (2015) studied the stability of EGFR mAb (rabbit anti-mouse IgG) with various concentrations of [Chol][Dhp]. EGFR mAb was incubated at 37 °C with protein kinase K and 20% or 50% w/w [Chol][Dhp]. In the IL-free sample, the protein was completely degraded in 1 h. In the 20% w/w IL sample, the protein exhibited fragmentation only after 12 h, indicating a slower degradation process. In the 50% w/w IL sample, the protein was not completely degraded even after 7 days and retained its α-helix structure. These results indicate that higher concentrations of ILs can suppress enzymatic degradation. Conformational stability was not significantly changed in both concentrations of ILs. Although the overall α-helix backbone of the protein remained unchanged, there were alterations in the folding pattern of certain regions. These structural changes could lead to differences in the aggregation behaviour of the protein, which was seen as the aggregation index increased from 1.03 to 1.23 for 20% w/w and 1.35 for 50% w/w [Chol][Dhp].

Dhiman et al. 2022 investigated the effect of [Chol][CH_3_COO], [Chol][Cl], [Chol][Dhc], and [Chol][Dhp] on IgG. All ILs at a concentration of 0.5–2.5 mM increased thermal stability in the order: [Chol][CH_3_COO] > [Chol][Cl] > [Chol][Dhc] > [Chol][Dhp] > IL-free. As the concentration of [Chol][CH_3_COO] and [Chol][Cl] increased, the melting temperature increased linearly up to 81 °C and 80 °C respectively, from the control of 76 °C. Despite the presence of extended hydrogen bonds in the [Chol][Dhc] and [Chol][Dhp] samples, none of the tested ILs caused significant alterations in the secondary structure of IgG. All ILs at these concentrations resulted in an increased amount of monomeric protein compared to the IL-free sample, suggesting that the conformational stability is increased. Long-term stability was assessed by storing the protein for 4 weeks in 2 mM and 2.5 mM of ILs. In all samples, the light and heavy chains remained unchanged after 4 weeks, indicating preserved stability. After 2 weeks, [Chol][Cl] was found to reduce the aggregation of the heavy chain more than the other samples. Counterintuitively, with [Chol][Cl], its secondary structures are more stable in 2 mM compared to 2.5 mM. Overall, every IL tested did not alter the protein secondary structure while increasing long-term and thermal stability. The most promising ILs for these applications were [Chol][CH_3_COO] and [Chol][Cl].

Shmool et al. (2021) assessed the stability of IgG4 with [Chol][Dhp] in bufferless solution and with two different excipient combinations. Formulation 1 contained 100 mg/mL IgG4, 34 mg/mL l-arginine HCl, 50 mg/mL trehalose dihydrate and 0.49 mg/mL polysorbate 20. Formulation 2 contained 50 mg/mL IgG4, 0.53 mg/mL l-histidine, 2.2 mg/mL l-histidine HCl, 25 mg/mL trehalose dihydrate and 0.20 mg/mL polysorbate 20. The melting temperatures were reduced by over 20 °C for every sample when 10% w/w [Chol][Dhp] was added to the bufferless solution and both formulations 1 and 2. It was also seen that formulation 2 created irreversible aggregates regardless of the presence of IL. Conformational stability was assessed using MD simulations. The structure of IgG4 in [Chol][Dhp] alone was altered significantly. This effect was significantly reversed when the excipients in formulation 1 were added. The movement of the Fab fragment in the presence of formulation 1 and [Chol][Dhp] is restricted, which indicates increased conformational stability. These results demonstrate that altering excipients with IL formulations can drastically change protein interactions.

Furthermore, Shmool et al. 2022) tested IgG4 in 10%, 30% and 50% w/w [Chol][Cl]. To investigate the effects of long-term storage conditions, both ILs and proteins were stored at 4 °C for a duration of 365 days. Generally, the increase in [Chol][Cl] concentration resulted in decreased pH and an increase in ionic strength, increasing conformational stability. This was observed as increasing IL concentration coincided with decreasing hydrodynamic radius in both the fresh and stored samples. The thermal stability was increased at higher IL concentrations in the fresh samples. The stability of the antibodies in 30% and 50% w/w ILs was close together with a melting temperature (Tm) of 67.8 °C and 69.2 °C, over the control at 63.2 °C. All the stored IL samples displayed similar Tm values to their fresh counterparts, while the non-IL sample saw a major drop in its Tm to 52.8 °C. This suggests that [Chol][Cl] is effective at preserving the structure of IgG4 over a long period of time.

## Discussion

Among the three classes of ILs studied with insulin and antibodies, choline-based ILs stand out as the promising stabilisers, showing potential for applications with both types of proteins. Studies using insulin aim to model the effects of ILs on small therapeutic proteins. These studies show that the optimal choice of IL cannot be generalised, as it is dependent on factors like IL concentration, insulin type, insulin concentration and buffer conditions. As a general trend, for choline aminoate ILs, a higher concentration enhances colloidal stability of insulin, but has potential to decrease conformational stability. Furthermore, an increased concentration of ILs demonstrates higher cytotoxicity. Therefore, future formulations will need to optimize IL concentration to achieve a balance among these factors.

Choline-based ILs with antibodies have been shown to enhance both colloidal and conformational stability while preserving antibody functionality over extended periods of storage. The effect of the IL is heavily dependent on its concentration and the presence of other excipients. Most of the studies utilised [Chol][Dhp]; however, other ILs will need to be investigated more extensively as they may have better stabilising properties. For example, [Chol][CH_3_COO] and [Chol][Cl] were shown to be better in long-term storage (Dhiman et al. 2022; Shmool et al. 2022). Furthermore, choline aminoate ILs has yet to be investigated with antibodies, although they have shown to be promising prospects with insulin, as previously discussed (Judy and Kishore 2023; Sundaram et al. 2022). Currently, there is very limited data available on commercially accessible therapeutic antibodies. Considering the promising results and the wide scope of mAbs in the protein space, further research in this field should be conducted.

Studies with alkylamine-based ILs showed strong performance in increasing both conformational and colloidal stability for insulin. However, it is important to note that these studies utilised a relatively high concentration of ILs to achieve these results. These ILs display higher toxicity associated with increasing chain length and most importantly increasing concentration (Kowalczyk et al. 2018; Ghosh et al. 2022; Piotrowska et al. 2017). While there is potential for it to be used as a stabiliser, it is essential to evaluate biocompatibility, particularly at lower concentrations for future formulations, as this may provide a better balance between enhancing stability and minimizing toxicity and viscosity.

Imidazolium-based ILs were mostly found to be denaturants. However, their mechanisms and interactions with proteins can still be extrapolated to other families of ILs. Through this, we have determined that protein-ion interactions are quite complex and may not always strictly adhere to the Hofmeister series. The Hofmeister series provides a general framework for understanding the relative effects of different ions on protein stability and solubility. But due to the intricate nature of protein interactions, it is necessary to develop more comprehensive models of protein aggregation considering factors beyond the simple classification of ions as kosmotropic or chaotropic.

Future directions to quantify these effects have increasingly shifted to molecular dynamics simulations. These simulations allow for high-resolution analysis of protein behaviour. However, it is also crucial that these simulations can be validated through physical experiments to ensure their accuracy and reliability.

Simulations probing the interactions of ILs with the protein surface are well quantified. Molecular dynamics simulations of choline aminoate ILs and insulin indicate that their stability is attributed to interactions involving the hydration shell around the protein, and the water-stripping effect of ions. The interactions are influenced to a greater extent by the anions (Sundaram et al. 2022). While ionic strength and hydrophobicity have been used to partially predict the effects of ILs, it is important to recognize that other factors, such as specific molecular interactions with the protein, also play a crucial role in determining stability (Shukla et al. 2011).

Thus, investigating the protein’s structural changes is an alternate strategy. This approach aims to quantify the conformational changes of proteins when exposed to these ions (Bui-Le et al. 2020; Ghanta et al. 2020). However, as demonstrated with choline-based ILs and insulin, conformational stability does not necessarily predict colloidal stability. Recently, advancements in computer hardware have enabled the modelling of full protein association pathways on timescales of seconds using Markov State Models (Plattner et al. 2017; Paul et al. 2017). More importantly, this method provides kinetic rates for transitions between different conformations, (Metzner et al. 2009) which can be validated through real-life experiments using techniques such as fluorescence. Currently, this approach is only applicable to small proteins and peptides, as larger proteins such as antibodies are too large to simulate effectively. However, we recommend keeping an eye on this area for future developments.

## Conclusion

ILs have shown great potential in enhancing the stability of therapeutic proteins, particularly insulin and mAbs. Research indicates that insulin formulations with choline aminoate and alkylamine ILs significantly improve colloidal stability; however, increasing concentrations of ILs may potentially decrease conformational stability and increase toxicity. The optimal choice of IL and its concentration differs based on the usage of the specific protein and buffer. Imidazolium ILs primarily act as denaturants, with the studies highlighting that predictions based solely on ionic strength are insufficient for determining protein stability. This underscores the need for more complex models to accurately predict protein aggregation mechanisms, especially in the presence of ILs.

Choline-based ILs have demonstrated a notable capacity to stabilise mAbs, especially when combined with other excipients. The dihydrogen phosphate anion, in particular, enhances thermal stability, while the chloride and acetate anions are beneficial for long-term storage. Despite the success of choline aminoates in stabilising small biologics like insulin, their potential in stabilising mAbs remains largely unexplored. Moreover, there is a significant lack of knowledge regarding the impact of ILs on commercially available mAbs. Given that mAbs represent the largest subset of protein drugs, it is crucial to understand how ILs affect their stability and functionality.

In summary, while ILs offer promising solutions for protein stabilisation, the optimal choice of IL and its concentration must be tailored to the specific protein and buffer system. Continued research is essential to fully harness the potential of ILs in therapeutic protein formulations.

## Data Availability

No datasets were generated or analysed during the current study.

## Abbreviations

IL: Ionic liquid
mAb: Monoclonal antibody
IG: Immunoglobulin
Fab: Fragment antigen binding
Fc: Fragment crystallizable
SOD: Superoxide dismutase
Chol: Choline
Gly: Glycinate
Leu: Leucinate
Val: Valinate
Pro: Prolinate
Asp: Aspartate
Glu: Glutamate
Arg: Argininate
Lys: Lysinate
CAGE: Choline geranate
BMIM: 1-Butyl-3-methylimidazolium
CH_3_COO: Acetate
CF_3_COO: Trifluoroacetate
C(CN)_3_: Tricyanomethaide
N(CN)_2_: Dicyanamide
SCN: Thiocyanate
Cl: Chloride
Br: Bromide
HSO_4_: Hydrogen sulfate
I: Iodide
TMAS: Trimethylamine sulfate
TEAS: Triethylamine sulfate
TMAP: Trimethylamine phosphate
TEAP: Triethylamine phosphate
TMAA: Trimethylamine acetate
EAN: Ethyl ammonium nitrate
PAN: Propyl ammonium nitrate
NO_3_: Nitrate
Dhp: Dihydrogen phosphate
KCl: Potassium chloride
HCl: Hydrogen chloride
Tris: Tris(hydroxymethyl)aminomethane
NaCl: Sodium chloride
DCl: Deuterium chloride
